# Machine learning in medicine: Addressing ethical challenges

**DOI:** 10.1371/journal.pmed.1002689

**Published:** 2018-11-06

**Authors:** Effy Vayena, Alessandro Blasimme, I. Glenn Cohen

**Affiliations:** 1 Health Ethics and Policy Lab, Department of Health Sciences and Technology, ETH Zurich, Zurich, Switzerland; 2 Harvard Law School, Cambridge, Massachusetts, United States of America

## Abstract

Effy Vayena and colleagues argue that machine learning in medicine must offer data protection, algorithmic transparency, and accountability to earn the trust of patients and clinicians.

A recent United Kingdom survey reports that 63% of the adult population is uncomfortable with allowing personal data to be used to improve healthcare and is unfavorable to artificial intelligence (AI) systems replacing doctors and nurses in tasks they usually perform [1]. Another study, conducted in Germany, found that medical students—the doctors of tomorrow—overwhelmingly buy into the promise of AI to improve medicine (83%) but are more skeptical that it will establish conclusive diagnoses in, for instance, imaging exams (56% disagree) [2]. When asked about the prospects of AI, United States decision-makers at healthcare organizations are confident that it will improve medicine, but roughly half of them think it will produce fatal errors, will not work properly, and will not meet currently hyped expectations [3]. These survey data resonate to the ethical and regulatory challenges that surround AI in healthcare, particularly privacy, data fairness, accountability, transparency, and liability. Successfully addressing these will foster the future of machine learning in medicine (MLm) and its positive impact on healthcare. Ethical and regulatory concerns about MLm can be grouped into three broad categories according to how and where they emerge, namely, around the sources of data needed for MLm and the approaches used in the development and deployment of MLm in clinical practice (for a hypothetical case study, see Fig 1).

**Fig 1:**
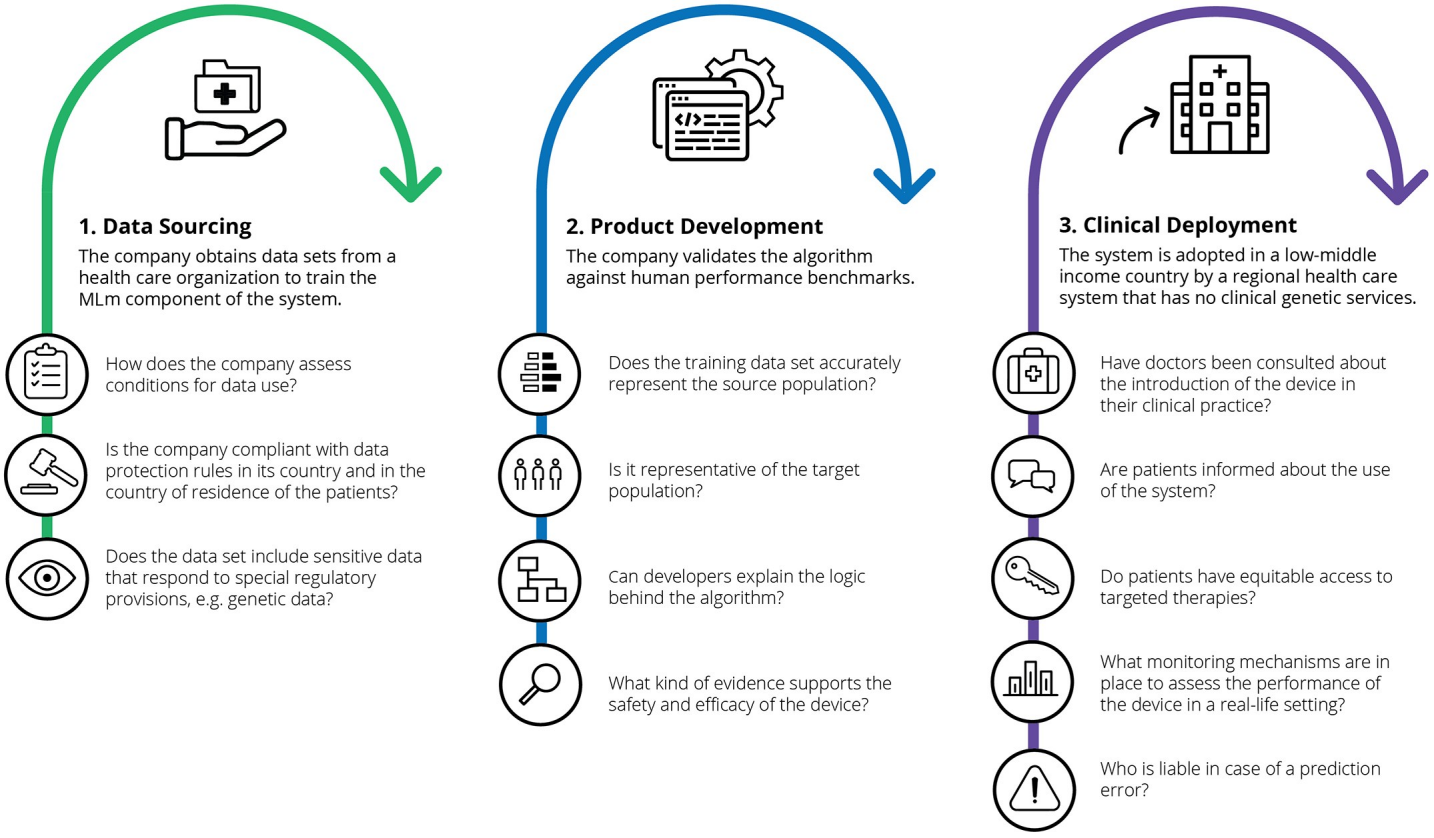
Imagine a medical software company developing a machine learning–based device. The device performs fully automated analysis of histopathology slides from cancer patients and predicts genetic mutations in tumors solely based on these images. This inferred genetic information can be used either for prognostic purposes or to detect an indication for a targeted therapy. Users will not know which features of the images the algorithm associates with mutated genes or the biological explanation for these associations. The selling propositions of the device are that it can infer valuable genetic information early in the diagnostic process and be used in contexts in which genetic testing is not available by analyzing images shared by pathologists on a cloud-based platform. MLm, machine learning in medicine.

## Data sourcing for MLm must adhere to data protection and privacy requirements

MLm algorithms use data that are subject to privacy protections, requiring that developers pay close attention to ethical and regulatory restrictions at each stage of data processing. Data provenance and consent for use and reuse are of particular importance [4,5], especially for MLm that requires considerable amounts and large varieties of data. It is very likely that such disparate data will have different conditions of use and/or be bound by different legal protections. A prominent example is the newly enacted European General Data Protection Regulation (GDPR), which sets out specific informed consent requirements for data uses and grants data subjects several rights that must be respected by those processing their data [6]. Moreover, this law applies to data from residents of the European Union (EU) irrespective of where the data are processed. Data that are used to train algorithms must have the necessary use authorizations, but determining which data uses are permitted for a given purpose is not an easy feat. This will also depend on data type, jurisdiction, purpose of use, and oversight models.

Ethical and regulatory concerns around health data have been debated for some time, and regulation such as GDPR is emblematic of legislative motivation to address novel digital challenges. However, different jurisdictions have reached and can be expected to reach different conclusions. Compare, for example, the GDPR with the Health Insurance Portability and Accountability Act [HIPAA] in the US, which focuses on healthcare data from patient records but does not cover other sources of health data such as health data generated outside of covered entities and business associates, e.g., data generated by life insurance companies or by a blood glucose–monitoring smartphone app [7].

## MLm development should be committed to fairness

The computer science adage goes, “garbage in, garbage out.” This is especially true for MLm, since the data sets on which MLm models are trained and validated are essential in ensuring the ethical use of predictive algorithms. Poorly representative training data sets can introduce biases into MLm-trained algorithms. “Bias” is a fraught term, with at least two archetypes common in medical data. First are cases in which the data sources themselves do not reflect true epidemiology within a given demographic, as for instance in population data biased by the entrenched overdiagnosis of schizophrenia in African Americans [8]. Second are cases in which an algorithm is trained on a data set that does not contain enough members of a given demographic—for instance, an algorithm trained mostly on data from older white men. Such an algorithm would make poor predictions, for example, among younger black women. If algorithms trained on data sets with these characteristics are adopted in healthcare, they have the potential to exacerbate health disparities [9].

To avoid this pitfall, scientific societies and regulatory agencies must develop best practices for recognizing and minimizing the downstream effects of biased training data sets, while bodies such as institutional review boards, ethics review committees, and health technology assessment organizations should check for compliance with such standards. As an example of one opportunity to reduce the risks of biased algorithms, the US Food and Drug Administration (FDA), in the context of its Digital Health Innovation Action Plan, has started a precertification pilot program to assess medical software under development based on five excellence criteria, including quality [10]. To avert the harms of biased training sets, the FDA quality criterion—and other regulatory criteria like it—could be expanded to cover the risk of bias in training data sets. This need is being addressed in some circles; for instance, the recent American Medical Association (AMA) policy recommendations on AI (in this case, “augmented intelligence”) promote the identification and minimization of biases in data [11]. A different approach, exemplified by the All of Us precision medicine research cohort in the US, is to fund the development of more representative data sets that can be used for training and validation [12].

In addition to regulatory protections against algorithmic bias, modernized regulatory approval—in the form of ad hoc guidance—is needed to maintain the safety and efficacy of MLm-based algorithms. These algorithms can in principle improve continuously with each use, resulting in real-time “updates” that cannot possibly be tested individually in clinical trials or assessed according to the typical timetable of a healthcare regulator. Regulators must develop standard procedures including effective postmarket monitoring mechanisms by which developers can transparently document the evolution of their MLm software.

## The deployment of MLm should satisfy transparency standards

Perhaps the MLm raising the most difficult ethical and legal questions—and the greatest challenge to current modes of regulation—is represented by noninterpretable, so-called black-box algorithms, the inner logic of which remains hidden even to their developers. This lack of transparency can preclude the mechanistic interpretation of MLm-based assessments and, in turn, reduce their trustworthiness. Moreover, the disclosure of basic yet meaningful details about medical treatment to patients—a fundamental tenet of medical ethics—requires that the doctors themselves grasp at least the fundamental inner workings of the devices they use. Therefore, for MLm to be ethical, developers must communicate to their end users—doctors—the general logic behind MLm-based decisions. Some degree of explainability may also be required to justify the clinical validation of MLm in prospective studies and randomized clinical trials. In the case of fully automated medical decisions, the level of risk associated with the procedure may determine whether and how information should be provided to patients about the presence of MLm-based technologies employed to guide their care. Communicating with patients about the use of MLm technologies may increase their trust and acceptance, which the survey data discussed above suggest is an ongoing concern.

As more diagnostic and therapeutic interventions become based on MLm, the autonomy of patients in decisional processes about their health and the possibility of shared decision-making may be undermined. This would happen, for instance, if reliance on automated decision-making tools reduces the opportunity of meaningful dialogue between healthcare providers and patients or if payers consider MLm recommendations as a precondition for reimbursement and refuse to cover treatments when the MLm recommends against them. Given the importance of personal contact and interaction between patients, healthcare professionals, and caregivers, it is key for healthcare providers to embed MLm in supportive and empowering delivery systems. Third-party auditing may also prove necessary, and such ethical integration may become a precondition for accreditation.

Special attention should be paid to the AI-enabled consumer informatics such as smartphone apps and wearable devices. Rapid progress in machine learning could result in a proliferation of health-related consumer products for which quality standards and accreditation systems should be developed.

Finally, the allocation and grounds for liability for adverse events related to the use of MLm will need to be clarified. Without a thorough ability to predict how various kinds of MLm will expose hospitals, physicians, and developers to liability, it will be hard to achieve widespread adoption [13,14].

## Conclusions

The clinical use of MLm may transform existing modes of healthcare delivery. MLm will be used in the clinical setting by healthcare professionals, be embedded in smart devices through the internet of things, and be used by patients themselves beyond the clinical setting for disease self-management of chronic conditions. The exponential growth of investment in MLm signals that research is accelerating, and more products may soon be targeting market entry. To merit the trust of patients and adoption by providers, MLm must fully align with data protection requirements, minimize the effects of bias, be effectively regulated, and achieve transparency. Addressing such ethical and regulatory issues as soon as possible is essential for avoiding unnecessary risks and pitfalls that will hinder further progress of MLm.

## Abbreviations

AI: artificial intelligence
AMA: American Medical Association
EU: European Union
FDA: Food and Drug Administration
GDPR: General Data Protection Regulation
HIPAA: Health Insurance Portability and Accountability Act
MLm: machine learning in medicine

## References

[1] Fenech M, Strukelj N, Buston O. Ethical, social and polictical challenges of artificial intelligence in health. 2018 April [Cited 19 Sept 2018]. http://futureadvocacy.com/wp-content/uploads/2018/04/1804_26_FA_ETHICS_08-DIGITAL.pdf.

[2] Pinto dos Santos D, Giese D, Brodehl S, Chon SH, Staab W et al Medical students’ attitude towards artificial intelligence: a multicentre survey. Eur Radiol 2018 7 6 10.1007/s00330-018-5601-1 29980928

[3] Intel Corporation. Overcoming barriers in AI adoption in healthcare. 2018 April [Cited Sept 19, 2018]. https://newsroom.intel.com/wp-content/uploads/sites/11/2018/07/healthcare-iot-infographic.pdf.

[4] Vayena E, Blasimme A. Biomedical big data: new models of control over access, use and governance. J Bioeth Inq. 2017;14:501–513. 10.1007/s11673-017-9809-6 28983835 PMC5715037

[5] Vayena E, Blasimme A. Health research with big data: Time for systemic oversight. The Journal of Law, Medicine & Ethics. 2018; 46: 119–129. 10.1177/1073110518766026 PMC6052857 30034208

[6] McCall B. What does the GDPR means for the medical community? The Lancet. 2018; 391: 1249–50. 10.1016/S0140-6736(18)30739-6 29619949

[7] Cohen IG, Mello MM. HIPAA and protecting health information in the 21st Century. JAMA. 2018;320(3):231–232. 10.1001/jama.2018.5630 29800120

[8] Neighbors HW, Jackson JS, Campbell L, Williams D. The influence of racial factors on psychiatric diagnosis: a review and suggestions for research. Community Ment Health J. 1989;25: 301–11. 2697490 10.1007/BF00755677

[9] Braveman P. Health disparities and health equity: concepts and measurement. Annu Rev Public Health. 2006;27:167–94. 10.1146/annurev.publhealth.27.021405.102103 16533114

[10] U.S. Food and Drug Administration. Developing Software Precertification Program: A Working Model. 2018 June [Cited 19 Sept 2018]. https://www.fda.gov/downloads/MedicalDevices/DigitalHealth/DigitalHealthPreCertProgram/UCM611103.pdf.

[11] The American Medical Association. AMA passes first policy recommendations on augmented intelligence. 2018 Jun 14 [Cited 19 Sept 2018]. https://www.ama-assn.org/ama-passes-first-policy-recommendations-augmented-intelligence.

[12] National Institutes of Health. All of Us research program. [Cited 28 Sept 2018]. https://allofus.nih.gov/

[13] Price WN. Regulating black-box medicine. Mich L Rev. 2017; 116(1): 421–74. 29240330

[14] Price WN. Medical Malpractice and Black-Box Medicine In Cohen I, Lynch H, Vayena E, Gasser U. (Eds.). (2018). Big Data, Health Law, and Bioethics. Cambridge, UK: Cambridge University Press.

